# The regulation of BK channel activity by pre- and post-translational modifications

**DOI:** 10.3389/fphys.2014.00316

**Published:** 2014-08-22

**Authors:** Barry D. Kyle, Andrew P. Braun

**Affiliations:** Department of Physiology and Pharmacology, Cumming School of Medicine, Libin Cardiovascular Research Institute, University of CalgaryCalgary, AB, Canada

**Keywords:** calcium-activated K^+^ channel, β subunit, phosphorylation, modulation, smooth muscle, neuron, contractility

## Abstract

Large conductance, Ca^2+^-activated K^+^ (BK) channels represent an important pathway for the outward flux of K^+^ ions from the intracellular compartment in response to membrane depolarization, and/or an elevation in cytosolic free [Ca^2+^]. They are functionally expressed in a range of mammalian tissues (e.g., nerve and smooth muscles), where they can either enhance or dampen membrane excitability. The diversity of BK channel activity results from the considerable alternative mRNA splicing and post-translational modification (e.g., phosphorylation) of key domains within the pore-forming α subunit of the channel complex. Most of these modifications are regulated by distinct upstream cell signaling pathways that influence the structure and/or gating properties of the holo-channel and ultimately, cellular function. The channel complex may also contain auxiliary subunits that further affect channel gating and behavior, often in a tissue-specific manner. Recent studies in human and animal models have provided strong evidence that abnormal BK channel expression/function contributes to a range of pathologies in nerve and smooth muscle. By targeting the upstream regulatory events modulating BK channel behavior, it may be possible to therapeutically intervene and alter BK channel expression/function in a beneficial manner.

## Introduction: BK channel distribution and architecture

BK channels, also called MaxiK/Slo1/K_Ca_1.1 channels, are a class of K^+^ ion channels that undergo extensive pre- and post-translational modification. BK channel α subunits are encoded by the *KCNMA1* gene, also known as *SLO*, and are ubiquitously expressed throughout mammalian tissues (e.g., neurons, smooth and skeletal muscles, exocrine cells). BK channels are assembled and strategically positioned on membrane surfaces, including the plasma membrane (Latorre et al., 1989), mitochondria and nucleus (Singh et al., 2012). Functional BK channels are multimeric structures composed of four similar pore-forming α subunits (Shen et al., 1994) and up to four regulatory β subunits can co-assemble with the tetrameric α subunit complex. The synergistic activation of BK channels by Ca^2+^ ions and depolarization causes a substantial K^+^ current that exhibits a large or “big” single channel conductance (i.e., up to 250 pS under symmetric K^+^ conditions). Activation of this formidable ionic current serves to drive membrane potential in the negative direction.

The transmembrane portion of the BK channel α subunit structure is thought to largely resemble that of voltage-gated K^+^ (K_v_) channel subunits in terms of voltage-sensing and pore-forming domains. Notably, BKα subunits contain an additional transmembrane segment, termed S0, resulting in an extracellular N-terminus. Specialized charged residues are present within the transmembrane segments S2–S4 of the BKα subunit that contribute to its voltage-sensing properties. While topologically similar to their K_v_ channel counterparts, BK channels display weaker or less sensitive voltage-dependent activation (i.e., the ionic conductance-voltage relation is less steep), due to an altered distribution of voltage-sensing residues within the S2–S4 segments (Ma et al., 2006). Mechanistically, membrane depolarization drives conformational re-arrangements in the voltage sensor domains, resulting in an upward twisting of the S4 segment relative to the pore domain; these conformational movements are reversed upon repolarization (Hoshi et al., 2013).

The C-terminal domain of the BKα subunit contains a considerable range of specialized structures that regulate channel function. These include several binding sites for divalent cations (i.e., Ca^2+^ and Mg^2+^) and regions that undergo dynamic post-translational modification such as phosphorylation. Each mammalian BKα subunit contains two “regulators of K^+^ conductance” (RCK) domains, arranged in tandem along the C-terminus; in the tetrameric channel complex, these RCK domains co-assemble to form an octomeric gating ring structure in the cytosol (Yuan et al., 2010). The RCK domains also have Ca^2+^-binding regions and are crucial in conferring the channel's Ca^2+^ ion sensing properties (Cui et al., 2009). Ca^2+^ ions bind to these specialized regions within the BKα C-terminus, leading to a structural expansion of the intracellular region of the ion conduction pathway that facilitates gating and K^+^ efflux (Yuan et al., 2012; Hoshi et al., 2013).

## Genetic diversity and splice variants

Unlike the K_v_ channel superfamily, which uses different genes to increase its genetic diversity, BK channels derive functional diversity through the alternative post-transcriptional splicing of mRNA derived from the single *KCNMA1* gene encoding the BKα subunit (Shipston, 2001). Up to ten distinct splice sites have been described in *KCNMA1* (Poulsen et al., 2009), leading to the generation of BKα subunits with different phenotypes and various functional roles, including altered sensitivity to Ca^2+^ and/or voltage (Shipston, 2001; Johnson et al., 2011), responses to phosphorylation (Tian et al., 2001), signaling cascades (Schubert and Nelson, 2001; Tian et al., 2001, 2004), membrane expression regulation (Alioua et al., 2008; Ahrendt et al., 2014), trafficking and lipidation (Toro et al., 2006; Zarei et al., 2007; Shipston, 2014). The impressive range of phenotypic products that can result from differential splicing of the *KCNMA1* gene product contributes to diversity of BK channel function between tissues, cells and intracellular compartments.

## BK channel auxiliary subunits

**Figure 1:**
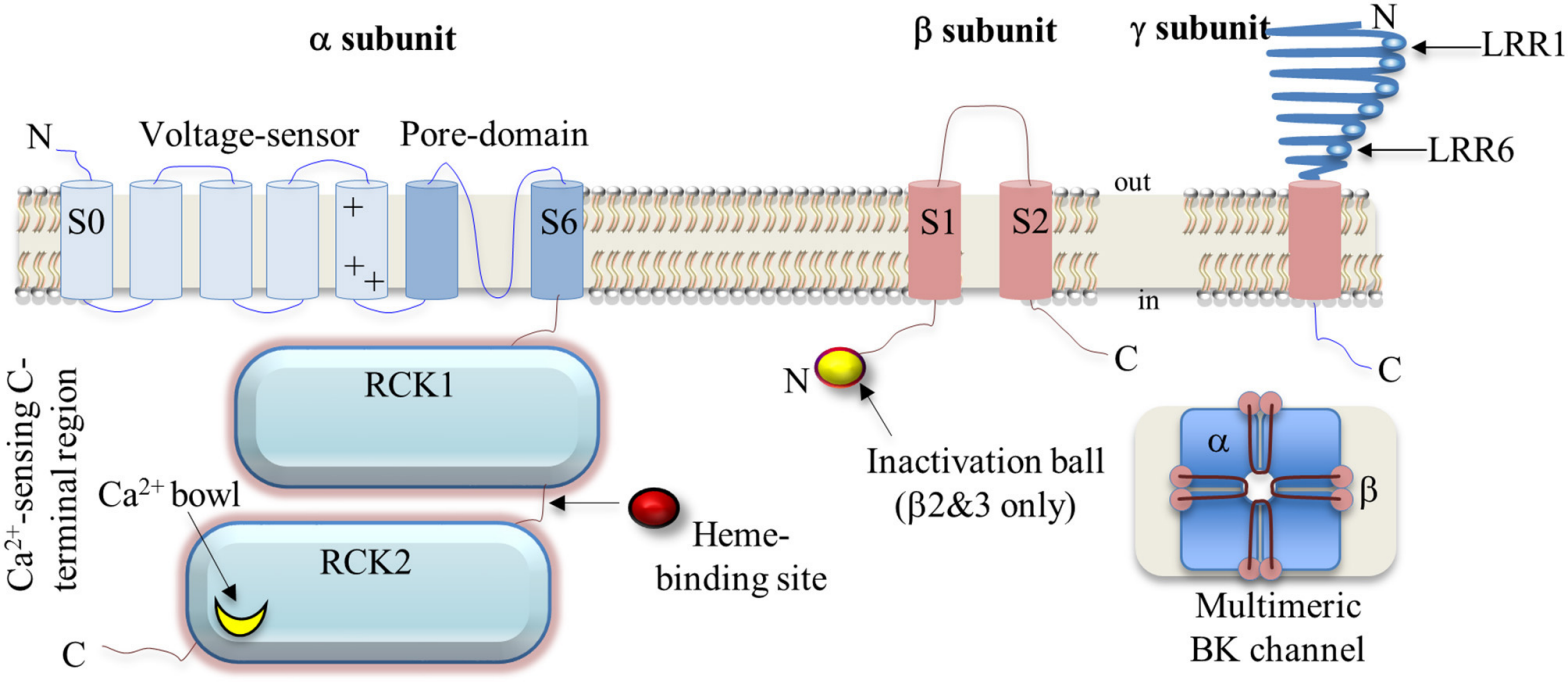
**A schematic illustration of BK channel α, β and γ subunit architecture with major structures defined**. Abbreviations: N, amino-terminus; C, carboxy-terminus; LRR, leucine-rich repeat; S, transmembrane segment; RCK, regulator of K^+^ conductance.

BK channels can co-assemble with modulatory auxiliary subunits BKβ 1-4 (Knaus et al., 1994a; Tanaka et al., 1997; Brenner et al., 2000a; Uebele et al., 2000), as well as a newly defined family of leucine-rich repeat containing subunits (LRRCs), referred to as γ subunits (Yan and Aldrich, 2010, 2012). Both BKβ and γ subunits contain sizeable extracellular regions and it is thought that these regions physically interact with the membrane-spanning domains of the BKα subunit. In particular, BKβ subunits appear to interact mainly with the N-terminal S0–S2 segments of the pore-forming BKα subunit (Morrow et al., 2006; Liu et al., 2008; Morera et al., 2012), thereby regulating channel opening through allosteric effects on the intramolecular processes underlying Ca^2+^ and/or voltage-dependent activation. As these auxiliary subunits are expressed in a tissue-specific manner, they confer distinct functional consequences by impacting BK channel kinetics and gating behavior. For instance, BKβ 1 subunits are typically expressed in smooth muscle, whereas BKβ 4 are expressed in neural tissue. BKβ subunits 1, 2 and 4 are reported to stabilize the channel's voltage sensor domains in the active conformation (Contreras et al., 2012), thereby enhancing channel activity, In contrast, BKβ 2 and β 3 subunits confer BK channel inactivation via an N-terminal “inactivation ball” (Wallner et al., 1999; Brenner et al., 2000a; Uebele et al., 2000) (Figure 1), which will limit K^+^ efflux and membrane hyperpolarization. To date, two functionally-distinct BKβ 2 splice variants (BKβ 2_a−b_) have been described in mammals, although BKβ 2_b_ does not appear to inactivate the channel complex (Ohya et al., 2010). Similarly, four functionally-distinct BKβ 3 splice variants (BKβ 3_a−d_) are known, with splice variants A-C conferring partial inactivation of BK channel current (Uebele et al., 2000). BKβ 4 subunits are the most distantly-related of the β subunits in terms of sequence similarity and produce mixed effects on BK channel gating, depending on the local Ca^2+^ concentration. At low Ca^2+^ concentrations, BKβ 4 appears to decrease channel activation, but at high Ca^2+^ concentrations, activation is enhanced (Brenner et al., 2000a; Wang et al., 2006).

The molecular mechanisms by which γ-subunits interact with and influence BK channel gating and kinetics are currently an area of active investigation. All four known LRRC proteins (i.e., LRRC26, 38, 52, and 55) have been reported to enhance voltage-dependent activation of BK channels (Yan and Aldrich, 2010, 2012), with LRRC26 producing an impressive shift of up to −150 mV.

## Role of BK channels in smooth muscle function and disease

**Figure 2:**
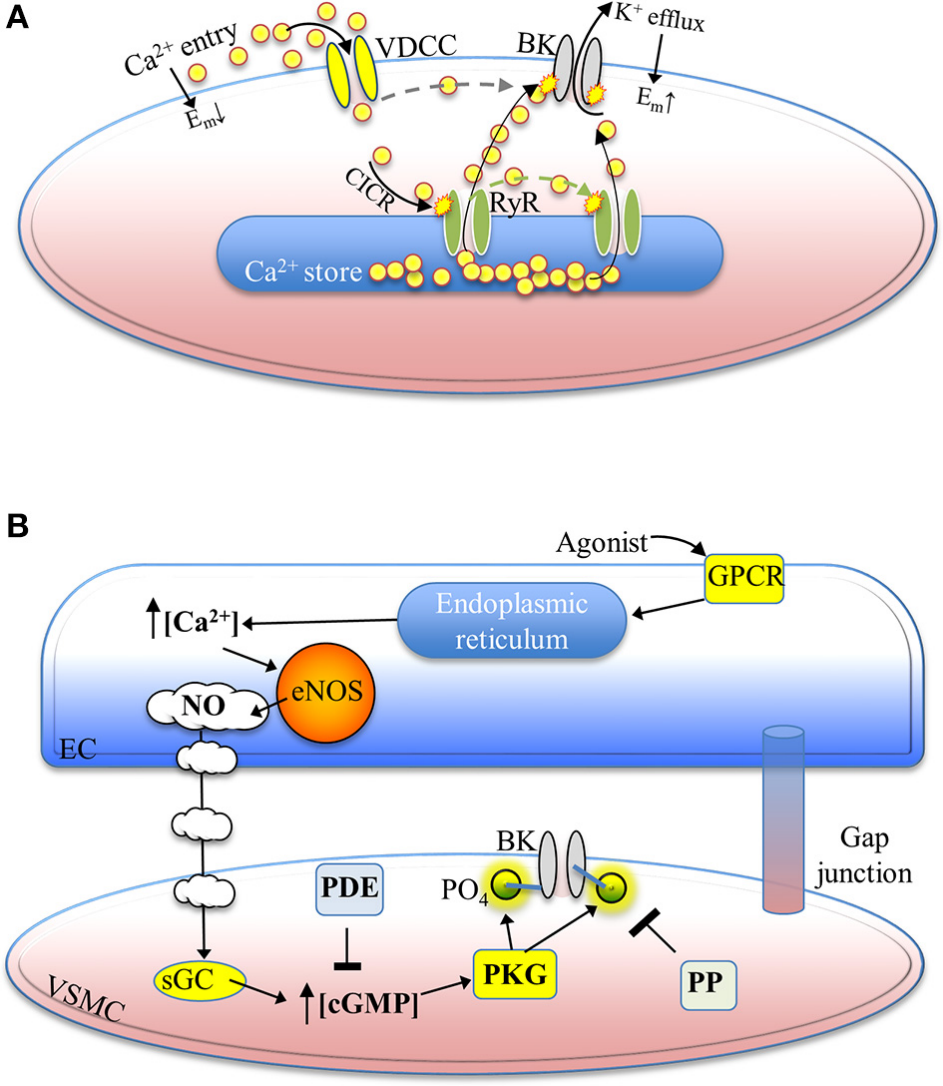
**A summary of select physiological mechanisms leading to BK channel activation and reversible phosphorylation-mediated enhancement**. **(A)** Ca^2+^ –dependent activation of BK channels hyperpolarizes the membrane potential. Depolarization of the membrane potential activates voltage-dependent Ca^2+^ channels, leading to Ca^2+^ entry and Ca^2+^-induced Ca^2+^ release from nearby ryanodine receptors. Released Ca^2+^ promotes BK channel activation, which drives the membrane potential in the negative (hyperpolarized) direction. Ca^2+^ influx via VDCCs may also contribute directly to BK channel activation (dotted line) as a result of the spatial proximity of these two channels within membrane nano/micro-domains. **(B)** Mechanisms underlying the generation of nitric oxide from an endothelial cell, with the NO/cGMP/PKG-mediated phosphorylation of a BK channel illustrated in an adjacent vascular smooth muscle cell. Nitric oxide release from endothelial cells binds to soluble guanylyl cyclase in smooth muscle cells, resulting in elevated intracellular cGMP concentrations. PKG is then activated and phosphorylates the BKα subunit. Phosphodiesterase activity lowers intracellular cGMP and protein phosphatase activity removes the regulatory phosphate from Ser/Thr residues of the BK channel protein. Abbreviations: VDCC, voltage-dependent Ca^2+^ channel; BK, BK channel; E_m_, membrane potential; CICR, Ca^2+^-induced Ca^2+^ release; RyR, ryanodine receptor; GPCR, GTP-binding protein-coupled receptor; eNOS, endothelial nitric oxide synthase; NO, nitric oxide; EC, endothelial cell; sGC, soluble guanylyl cyclase; PDE, phosphodiesterase; PO_4_, phosphate group; cGMP, cyclic guanosine monophosphate; PKG, protein kinase G; PP, protein phosphatase; VSMC, vascular smooth muscle cell.

Phasic smooth muscles, such as those lining the urinary bladder, urethra and ureters, undergo action potential (AP) events, with rapid depolarization-repolarization fluctuations. APs cause a significant global increase in intracellular [Ca^2+^] and BK channels are largely responsible for the rapid down-stroke (repolarization) phase (Burdyga and Wray, 2005; Thorneloe and Nelson, 2005; Kyle et al., 2013b). In contrast, tonic smooth muscles, such as those found throughout vascular tissue and much of the gastrointestinal tract and airways, regulate lower magnitude changes in membrane potential by principally responding to localized elevations in intracellular [Ca^2+^] mediated by ryanodine receptors (RyRs) (Figure 2). The dynamic post-translational “tuning” of BK channels permits considerable diversity in the biophysical properties of the current.

In common with many other tetrameric K^+^ channels in smooth muscles, the amplitude of K^+^ current carried through BK channels in smooth muscles can be dynamically regulated by post-translational modifications to the channel complex, including the reversible phosphorylation of the pore-forming BKα subunit by a number of protein kinases, as described below. Almost all phosphorylation sites are conserved in mammalian BK channel splice variants.

Many tissues have distinct macromolecular signaling complexes underlying the function of ion channels. Smooth muscles, for instance, generally have closely-associated RyRs, which periodically release Ca^2+^ and cause local elevations in [Ca^2+^]_i_ (i.e., 10–20 μM) (Pérez et al., 1999; ZhuGe et al., 2002) near BK channels positioned on the plasma membrane, which is sufficient to significantly raise the P_o_ and efflux K^+^ (Figure 2). The RyRs themselves are often close to Ca^2+^ influx pathways, for instance voltage-gated Ca^2+^ channels, or in proximity to IP_3_ receptors (Ohi et al., 2001).

The primary role of BK channels in vascular smooth muscle (VSM) is to repolarize/hyperpolarize the cell membrane potential in the face of chronic depolarizing stimuli, thereby reducing contractile activity. It is now well-recognized that enhancement of BK channel current in VSM via phosphorylation is principally-regulated by nitric oxide (NO)/cGMP/PKG signaling (Feil et al., 2003) (see Section BK Channel Modulation via Protein Phosphorylation below). NO is a gaseous second messenger synthesized mainly by the adjacent endothelial cell layer lining the lumen of all blood vessels (Fleming and Busse, 2003). Therefore, BK channel activity is considered to be closely linked with endothelial cell activity. Therapeutically, NO and synthetic NO donors are used to treat a range of vascular disorders, including angina pectoris and hypertension (Wimalawansa, 2008).

In addition to the urinary tract and VSM, BK channels are also important regulators in mediating the proper function of various other smooth muscles, including those found in the gastrointestinal tract, airway, and uterus. Their function, however, varies between cell types and layers, and generally is dependent on the associated macromolecular signaling complex. In the colon, for instance, BK channels contribute to setting the resting membrane potential in longitudinal smooth muscle, whereas in the circular layer, they limit excitatory responses (Sanders, 2008).

In VSM, a single amino acid polymorphism in the BKβ 1 subunit (i.e., E65K) is reported to have a gain-of-function effect on BKs channel activation and has been associated with lower systolic and diastolic blood pressures and a decreased prevalence of diabetic hypertension in humans (Fernández-Fernández et al., 2004; Nielsen et al., 2008). In contrast, BKβ 1 subunit expression is decreased in some forms of genetic hypertension (Amberg and Santana, 2003). Moreover, a point mutation (R140W) in the BKβ 1 subunit that modestly impairs channel opening has been linked with asthma severity in African-American males (Seibold et al., 2008). Provocative data from Jaggar and colleagues further suggest that the majority of BKβ 1 subunits reside within the cell interior and assemble with α subunits at the cell surface in a dynamic fashion (Leo et al., 2014). NO signaling appears to promote the forward trafficking of internal BKβ 1 subunits to the cell membrane, where they co-associate with BKα subunits to enhance channel activation. The authors suggest that auxiliary BKβ 1 subunits undergo selective endocytosis from the plasma membrane, followed by re-insertion in response to a vasodilatory stimulus, such as NO. These data imply that native BK channels in VSM may not always contain a full complement of β 1 subunits (i.e., the ratio of β 1 to α subunits in a single channel complex is <1), as described in rat cremaster artery (Yang et al., 2009), and that the subunit stoichiometry of these channels is not permanent. Dynamic regulation of BK channel subunit co-assembly and interaction at the plasma membrane may thus represent a novel paradigm for the modulation of ion channel activity.

Many research groups have reported that BK channel activity is upregulated during hypertension, and its contribution is apparently enhanced compared to normotensive animals (for review, see Joseph et al., 2013). It should be noted, however, that downregulation of BK channel activity has also been reported during hypertension (Amberg et al., 2003; Amberg and Santana, 2003; Nieves-Cintrón et al., 2007; Yang et al., 2013). Investigators have speculated that this decrease may be due to reduced BKβ 1 subunit expression/coupling, which would dampen the Ca^2+^ sensitivity of BK channel activation. Several research groups have reported that BK current density is positively-correlated to blood pressure in hypertensive animals (Rusch et al., 1992; England et al., 1993; Rusch and Runnells, 1994; Liu et al., 1998). Aortic smooth muscle isolated from rats with renal hypertension, spontaneously-hypertensive rats (SHR) and stroke-prone SHR (Rusch et al., 1992; England et al., 1993; Liu et al., 1998) exhibits significantly-upregulated BK channel activity, likely as a compensatory response. Collectively, these studies indicate that the expression and function of BK channels in the vasculature involves complex expression and signaling pathways, and may vary between cells, tissues, vascular beds and pathophysiological profiles.

BK channels are densely-expressed in mammalian bladder tissues (~20 channels per square micrometer) (Ohi et al., 2001) with BKβ 1 auxiliary subunits. BKα subunit knockout mice have demonstrated bladder dysfunction and exhibit a depolarized resting membrane potential in isolated bladder smooth muscle cells and intact tissues, indicating a role for BK channels in setting the membrane potential (Sprossmann et al., 2009). Inhibition of BK channel current with iberiotoxin in the bladders of healthy mice led to similar effects (Heppner et al., 1997; Hristov et al., 2011). BKβ 1-knockout mice similarly display overactive bladder symptoms, and a significant decrease in BK channel activity (Petkov et al., 2001). Intriguingly, bladder smooth muscle tissue taken from patients with neurogenic bladder over-activity exhibit little to no response to BK channel inhibition by iberiotoxin, or the channel agonist NS1619, indicating severe BK channel dysfunction (Oger et al., 2010). Macroscopic current recordings from these tissues demonstrated a significantly lower BK channel current density that mirrors that reported for experimentally-induced partial urethral obstruction in rats (Aydin et al., 2012). Patients with benign prostatic hyperplasia experiencing overactive bladder symptoms also demonstrate a parallel reduction in BK channel expression (Chang et al., 2010). Overexpression of BK channel protein in rats with experimentally-induced partial urethral obstruction proved to be an effective treatment for the existing overactive bladder activity (Christ and Hodges, 2006). These data collectively indicate that BK channels are important regulators of bladder smooth muscle excitability, and a potential target for therapeutic intervention for overactive bladder conditions.

## Role of BK channels in neuronal function/dysfunction

BK channels are abundantly expressed in both central and peripheral neurons, with prominent expression reported in both the cell body and pre-synaptic terminals (Faber and Sah, 2003). Functionally, these channels are key regulators of neuronal excitability, as channel opening will reduce action potential (AP) amplitude and duration, increase the magnitude of the fast after-hyperpolarization (fAHP) immediately following repolarization and limit the frequency of AP burst firing (Bielefeldt and Jackson, 1993; Faber and Sah, 2003; Gu et al., 2007; Haghdoost-Yazdi et al., 2008). At the pre-synaptic nerve terminal, localized BK channel activity can modulate both the amplitude and duration of depolarization-evoked Ca^2+^ entry as a result of the rapid repolarization and deactivation of voltage-gated Cav 2.1 (i.e., P/Q-type) and 2.2 (N-type) Ca^2+^ channels (Robitaille and Charlton, 1992; Issa and Hudspeth, 1994; Marrion and Tavalin, 1998; Fakler and Adelman, 2008). Reduced Ca^2+^ influx will limit vesicle fusion at active zones, leading to decreased neurotransmitter release (Roberts et al., 1990; Hu et al., 2001; Raffaelli et al., 2004).

Dissecting the functional roles of BK channels in the nervous system has been greatly aided by the availability of highly selective toxins (i.e., iberiotoxin) (Kaczorowski and Garcia, 1999) and small molecule inhibitors (e.g., penitrem A, paxilline, lolitrem B) (Knaus et al., 1994b; Imlach et al., 2008; Nardi and Olesen, 2008), along with the generation of genetically-engineered mice lacking either BKα or β subunits (Brenner et al., 2000b, 2005; Plüger et al., 2001; Meredith et al., 2004; Sausbier et al., 2004). Such strategies have revealed that the loss of neuronal BK current, either acutely or chronically, increases membrane excitability by decreasing the magnitude of the fAHP. Reducing the fAHP facilitates more rapid membrane depolarization in response to a tonic stimulus, leading to higher frequency AP firing. Such alterations in neuronal activity are typically associated with neurological disorders in the CNS, including tremor and ataxia (Sausbier et al., 2004; Brenner et al., 2005; Imlach et al., 2008). Interestingly, a point mutation in the RCK1 domain of the BKα subunit (i.e., D434G) identified in a subset of epileptic patients has been shown to increase neuronal BK channel activity by enhancing Ca^2+^-dependent channel gating (Du et al., 2005; Wang et al., 2009; Yang et al., 2010). Functionally, increasing BK activity and the associated fAHP may augment membrane excitability in the soma by enhancing the recovery rate of fast Na^+^ currents from voltage-dependent inactivation and reducing the absolute refractory period of neuronal firing.

In the CNS of mice and humans, genetic knockout or mutational disruption of the molecular chaperone cysteine string protein (CSPα) is linked with early onset neurodegeneration (Fernandez-Chacon et al., 2004; Donnelier and Braun, 2014), and interestingly, these conditions are associated with a significant up-regulation of BK channel expression in mouse brain and cultured neurons (Kyle et al., 2013a; Ahrendt et al., 2014). Although the mechanistic link between increased BK expression/activity and neurodegeneration remains undefined, it is hypothesized that increased BK current density in pre-synaptic terminals and/or the soma may lead to disrupted synaptic membrane excitability and neurotransmitter release. As described below, elevated BK channel expression in the CNS is closely linked with epilepsy, strongly suggesting that increased BK current density can lead to neurological disorders and possibly synaptic dysfunction/degeneration.

## Post-translational modification

Heteromeric BK channel complexes are the subject of extensive post-translational modifications, which can significantly alter channel behavior. Some modifications are highly-complex and require prior upstream modification(s) to the channel subunits.

### BK channel modulation via protein phosphorylation

Perhaps the most studied enzymatically-driven modification of BK channels is the addition of phosphate (PO^3−^_4_) groups to functionally-important residues (Ser/Thr/Tyr) present within the channel's pore-forming α subunit. These reactions are catalyzed by select protein kinases and are reversed by the actions of protein phosphatases that dephosphorylate these sites following removal of the stimulus. Phosphorylation can be either stimulatory or inhibitory with respect to the open probability of the channel and can depend on several variables (see below).

Regulation of BK channel activity in smooth muscles by phosphorylation-dependent signaling pathways is well documented (Schubert and Nelson, 2001) and the main modifying enzymes include cAMP- and cGMP-dependent protein kinases (i.e., PKA and PKG, respectively), protein kinase C (Zhou et al., 2010) along with c-Src tyrosine kinase (Davis et al., 2001). Biochemically, PKA is comprised of 2 catalytic and 2 regulatory subunits and kinase activation occurs in response to the direct binding of the second messenger cAMP to the regulatory subunits (Taylor et al., 1990). Cyclic AMP synthesis occurs following stimulation of adenylyl cyclase by hormones (e.g., adenosine, β-adrenergic agonists, PGI_2_, PGE_2_, etc.) or direct activators (e.g., forskolin). In the case of PKG activation, synthesis of cGMP can occur via a soluble or a membrane-bound form of guanylyl cyclase (Münzel et al., 2003); the former is typically activated by NO and the latter by natriuretic peptides acting on the cell surface receptors NPR-A and NPR-B. Structurally, PKG exists as a homodimer in which each monomer consists of a regulatory and catalytic domain linked in a single polypeptide chain (Francis et al., 2010); holo-PKG thus closely resembles the overall structure of PKA. Generally, PKA and PKG-mediated phosphorylation leads to BK channel enhancement, whereas PKC leads to channel inhibition. It should be stressed, however, that these regulatory effects on BK channel activity depend upon contextual phosphorylation/modification at multiple sites (Zhou et al., 2010, 2012; Kyle et al., 2013c), and may be further influenced by the constitutive phosphorylation status of the channel complex (see below). Selective blockade of the phosphodiesterase enzymes responsible for cGMP metabolism by pharmacologic agents such as sildenafil will prolong cGMP effects in smooth muscle and this process has been exploited therapeutically to treat erectile dysfunction and pulmonary hypertension (Francis et al., 2010). For a comprehensive overview of early studies describing BK channel regulation by kinase-associated pathways, see Schubert and Nelson (2001).

Using a multi-faceted strategy involving protein biochemistry, site-directed mutagenesis and patch clamp recordings, our group has recently reported that NO/cGMP/PKG signaling in VSM cells leads to the modification of three distinct Ser residues in the BKα C-terminus (i.e., Ser 691, 873 and 1111–1113), which directly correlate with enhancement of channel activity (Kyle et al., 2013c). Not unexpectedly, one of these sites (i.e., Ser873) is also important for PKA-mediated enhancement of BK activity (Nara et al., 1998). The regulatory phosphorylation status of BK channels also appears to differ developmentally, as BK channels in fetal arteries display more enhanced activity compared with channels from adult VSM (Lin et al., 2005, 2006). Augmentation of BK channel activity by NO/cGMP/PKG signaling is readily reversible and this is largely due to dephosphorylation via Ser/Thr protein phosphatases. Several studies have described involvement of protein phosphatases 1 and 2A in the regulation of BK channel activity, based mainly on the selective actions of inhibitors, such as okadaic acid (Zhou et al., 1996, 2010; Sansom et al., 1997).

Activation of PKC is reported to inhibit BK channel activity in VSM via the putative phosphorylation of Ser695 and Ser1151, and these modifications also appear to interfere with the stimulatory effects mediated by PKA and PKG (Zhou et al., 2010). Interestingly, this PKC-mediated inhibition of channel activity is absent in STREX-containing BKα splice variants (Zhou et al., 2012) (see below).

Similar to VSM, neuronal BK channel activity can be enhanced in response to regulatory phosphorylation of the pore-forming BKα subunit by both PKA and PKG, which can be reversed by the actions of Ser/Thr phosphatases 1 and 2A (Reinhart et al., 1991; Reinhart and Levitan, 1995; Sansom et al., 1997; Tian et al., 1998). Interestingly, proteomic analyses of rat brain BK channels isolated under basal conditions has identified ~30 Ser and Thr residues that appear to be constitutively phosphorylated *in vivo*, with 23 of these modified residues located within the channel's C-terminus (Yan et al., 2008). Such observations suggest that constitutive phosphorylation may help stabilize BK channel tertiary structure and/or create binding sites for interacting proteins. The various protein kinases responsible for these *in vivo* modifications are presently unknown, as is the extent to which channels from other tissues or expressed heterologously exhibit constitutive phosphorylation. Our recent data describing a role for multiple phosphorylation sites to support cGMP-dependent augmentation of BK channel activity in VSM cells (Kyle et al., 2013c) promote the idea that individual phospho-Ser/Thr residues act synergistically to enhance BK channel activity.

In neurons and neuroendocrine cells (e.g., pituitary, adrenal gland) and more recently in VSM (Nourian et al., 2014), a portion of BK channels identified by qRT-PCR contain the STREX splicing insert, a 59 amino acid insert present at splice site C2 within the C-terminus (Xie and McCobb, 1998; Shipston, 2001). In response to cAMP/PKA signaling, a Ser residue within the STREX insert can undergo phosphorylation, which has been shown to decrease BK channel activity (Tian et al., 2001). Functionally, such a change would be expected to enhance membrane excitability in neuroendocrine cells and promote exocytosis. Interestingly, phosphorylation of the STREX domain also appears to override the positive gating effects mediated by PKA-induced phosphorylation at other C-terminal sites, leading to an overall dominant-negative effect of STREX phosphorylation on BK channel activity (i.e., a single STREX-containing α subunit within a tetrameric channel is sufficient to flip PKA-mediated phosphorylation from stimulatory to inhibitory) (Tian et al., 2004). Furthermore, this inhibitory effect of PKA on BK channel activity appears to depend upon the presence of palmitoyl fatty acid groups within the STREX insert (Shipston, 2014), as palmitoylation-incompetent BK channels do not undergo PKA-mediated phosphorylation of the STREX insert and a decrease in activity (Tian et al., 2008). Collectively, these findings suggest that presence of STREX insert will lead to association of a C-terminal domain with the plasma membrane, which appears necessary for PKA-mediated phosphorylation within the STREX insert and inhibition of channel activity. Interestingly, presence of the STREX insert also appears to prevent the inhibitory effect of protein kinase C (PKC) on BK channel opening, possibly by inducing a conformation that precludes PKC-induced phosphorylation of Ser695 within the linker joining RCK1 and RCK2 domains (Zhou et al., 2012).

In addition to Ser/Thr phosphorylation, BK channels also undergo direct Tyr phosphorylation in the presence Src family kinases (i.e., c-Src and Hck) and the Ca^2+^-sensitive tyrosine kinase Pyk-2 (Ling et al., 2000, 2004; Alioua et al., 2002; Yang et al., 2012). Functionally, direct tyrosine phosphorylation of the BKα subunit has been reported to either increase (Ling et al., 2000, 2004; Yang et al., 2012) or decrease (Alioua et al., 2002) channel activity, although the reason(s) for this discrepancy remains unclear. Work from our group has shown that Phe substitution of Tyr766 in the C-terminus largely inhibits c-Src-induced BKα subunit phosphorylation, but does not appear to disrupt Pyk-2 mediated modification (Ling et al., 2000, 2004). Future studies examining the direct phosphorylation of native BK channels by tyrosine kinases *in situ* are needed to clarify the physiologic importance of this regulatory event.

### Endogenous regulatory molecules

Endogenous molecules (e.g., heme, carbon monoxide (CO), reactive oxygen species) have been reported to interact with the BK channel complex (for review, see Hou et al., 2009). Similarly, acidification of the cytosol (i.e., pH 6.5) is able to increase BK channel activation by left-shifting the voltage dependence by ~45 mV, but such effects can be readily masked by physiological levels of free Mg^2+^ (i.e., 1 mM) and Ca^2+^ (i.e., 1 μM) (Avdonin et al., 2003). The importance of [H^+^] with regards to BK channel activity may become more apparent during pathological conditions where fluctuations in [H^+^] and [Ca^2+^] may occur (e.g., cerebral ischemia) (Lipton, 1999).

The linker between the RCK1 and RCK2 regions of the BKα subunit (Figure 1) reportedly contains a binding site for intracellular heme molecules (Hou et al., 2009). Application of heme to the cytosolic face of BK channels was found to inhibit channel opening with an IC_50_ ~70 nM (Tang et al., 2003), likely via an allosteric process. Moreover, the direction of gating modulation by heme appears to be closely-linked to membrane potential, as BK channel P_o_ is enhanced at negative membrane potentials and inhibited at positive potentials. Heme regulators, transporters and degradation products (e.g., CO) are currently under investigation for their therapeutic potential in influencing BK channel activity and thus, global membrane potential (Hou et al., 2009).

Soluble guanylyl cyclase (sGC) contains an iron (heme) center that serves to bind NO, however, this site is also targeted by CO, which can activate sGC, leading to increased cytosolic [cGMP], PKG activation and enhanced BK channel activity (see Figure 2B). It has been further suggested that CO, along with NO, can also directly augment BK channel activity when applied at sufficiently-high concentrations (Hou et al., 2009; Leffler et al., 2011). Further examination of the physiologic contribution of such effects to BK channel regulation are warranted.

Reactive oxygen species (ROS) that are reported to influence BK channel behavior include hydrogen peroxide (H_2_O_2_), superoxide (O^−^_2_) and peroxynitrite (ONOO^−^). Increased levels of ROS may occur under localized conditions, such as atherosclerosis (Li and Förstermann, 2009) and are particularly troublesome, as H_2_O_2_ and O^−^_2_ will react with free NO to generate ONOO^−^, thereby reducing NO bioavailability and cGMP/PKG signaling in vascular smooth muscle. For detailed discussions on impact of ROS on BK channel activity, the reader is referred to excellent review articles (Tang et al., 2004; Hou et al., 2009).

### Regulation of BK channel expression by ubiquitination

Protein ubiquitination has emerged as a ubiquitous quality control mechanism for the regulation of protein trafficking and turnover and has been implicated in the dynamic control of diverse cellular processes (e.g., gene transcription, synaptic development and plasticity, oncogenesis, etc.) (Hershko and Ciechanover, 1998). Protein ubiquitination functions as a tagging system to mark proteins for degradation by the 26S proteasome complex and the human genome is reported to contain >600 genes encoding E3 ubiquitin ligases (Li et al., 2008), the enzyme responsible for conjugating ubiquitin monomers to target substrates. Given this level of abundance, the ubiquitin-proteasome system (UPS) appears to enzymatically parallel protein phosphorylation, for which ~520 putative kinase genes have been described (Manning et al., 2002), as a widespread mechanism for protein modification and the regulation of cellular function. Recent evidence indicates that BK channels also undergo ubiquitination, which appears to have important functional implications. In the CNS, interaction of BK channels with cereblon (Jo et al., 2005), a substrate receptor for the CRL4A E3 ligase, leads to ubiquitination of the BKα subunit and retention of modified channels in the endoplasmic reticulum (Liu et al., 2014). Preventing ubiquitination of BK channels by pharmacologic or genetic interference of the CRL4A enzyme complex leads to increased trafficking of BK channels to the neuronal cell membrane and a higher incidence of seizure induction and epilepsy in mice. Such data point to ubiquitination as an important quality control mechanism to limit BK channel expression in neurons, which will ultimately impact membrane excitability. Given that cereblon transcripts are also widely expressed in tissues outside the CNS, this regulatory paradigm may have broader functional importance. As noted above, disruption of the neuronal chaperone CSPα in mice also elevates BK channel expression, suggesting that increased channel density be a common contributing factor to excitation-related neuropathologies.

In VSM, BKβ 1 subunits are reported to undergo ubiquitination in cultured myocytes exposed to high glucose and in arteries obtained from mice made diabetic by injection of streptozotocin, a pancreatic β-cell poison. Diabetes-like conditions elevate the expression of a muscle-specific RING finger E3 ubiquitin ligase via enhanced NF-κ B transcriptional activity, leading to increased BKβ 1 subunit ubiquitination and proteolysis (Yi et al., 2014). As previously described, loss of the BKβ 1 subunit would be expected to decrease Ca^2+^- and voltage-dependent activation of VSM BK channels (Brenner et al., 2000b), leading to exaggerated membrane depolarization and smooth muscle contraction. As BKβ 1 subunits may be capable of dynamically assembling with BKα subunits at the membrane (Leo et al., 2014), ubiquitination of BKβ 1 alone may not necessarily result in a decreased cellular level of BKα subunits.

## Concluding remarks

**Figure 3:**
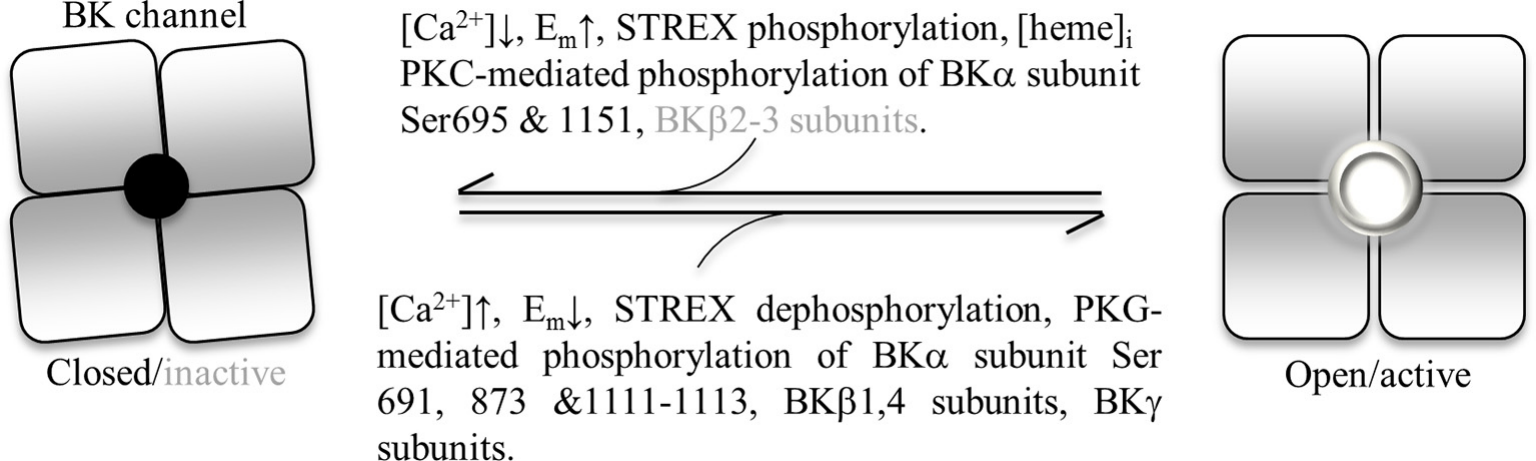
**A summary of cellular events/factors leading to BK channel activation (open pore) and deactivation/inactivation (closed pore)**. Abbreviations: E_m_, membrane potential; STREX, stress-axis regulated exon; PKC, protein kinase C; Ser, serine.

BK channel activity is regulated both directly and indirectly through a diverse range of modulatory pathways involving covalent modifications, metabolic factors, trafficking events and transcriptional processes (see Figure 3). Given the formidable effect that BK channels can exert on membrane excitability, as a result of their large single channel conduction and dual activation by membrane depolarization/cytosolic free Ca^2+^, such “fine-tuning” affords cells the ability to precisely control the impact of these channels on their function and responsiveness to both acute and chronic stimuli. As reinforced by the accompanying articles in this thematic issue, BK channels represent powerful effectors in tissue health and dysfunction and that understanding their modes of regulation may lead to novel therapeutic strategies in disease treatment.

### Conflict of interest statement

The authors declare that the research was conducted in the absence of any commercial or financial relationships that could be construed as a potential conflict of interest.

## Author note

Lingle and coworkers have demonstrated that the γ1 subunit (i.e., LRRC26) mediated leftward shift in BK channel gating occurs in an all-or-none fashion, in contrast to the incremental shifts in gating produced by stoichiometric association of BKβ1 subunits (Proc. Natl. Acad. Sci. U.S.A. 111, 4873, 2014. doi: 10.1073/pnas.1322123111). Subsequently, Evanson et al. (2014) have reported that LRRC26 is endogenously expressed in rat cerebral vascular myocytes and may function as an auxiliary γ1 subunit by altering the voltage and calcium sensitivity of BK channel gating (Circ. Res. 115, 423–431. doi: 10.1161/CIRCRESAHA.115.303407).
